# Clinical significance of long non-coding RNA NORAD in rheumatoid arthritis

**DOI:** 10.1186/s42358-024-00349-z

**Published:** 2024-01-18

**Authors:** Xueru Zhao, Weiyi Lin, Wenhui Zhou

**Affiliations:** 1 Department of Joint Surgery, Lishui People’s Hospital, No.1188, Liyang Road, 323000 Lishui, Zhejiang China; 2 Department of Emergency Medicine, Lishui Municipal Central Hospital, 323000 Lishui, Zhejiang China

**Keywords:** Rheumatoid arthritis, lncRNA NORAD, miR-204-5p, Diagnosis, Inflammatory factor

## Abstract

**Background:**

Rheumatoid arthritis (RA) is a chronic autoimmune disease that may cause joint deformities and seriously affect the normal life of the patients. In order to enable patients to receive timely attention and treatment, this study developed new diagnostic markers by exploring the expression and molecular mechanism of the long non-coding RNA NORAD (NORAD) in RA.

**Methods:**

Participants including 77 RA patients and 52 healthy persons were enrolled, and the corresponding clinical data and serum samples were obtained. The NORAD and miR-204-5p expression were detected by real-time fluorescence quantitative polymerase chain reaction (RT-qPCR). The content of inflammatory cytokines (IL-6, TNF-α) were determined through enzyme-linked immunosorbent assay (ELISA). Luciferase activity reporter assay demonstrated the association between NORAD and miR-204-5p. In addition, receiver operating characteristic (ROC) curve was used to evaluate the diagnostic efficacy of NORAD, and Pearson’s correlation analysis was applied for the correlation analysis.

**Results:**

NORAD was enriched in RA serum with high diagnostic value. Simultaneously, IL-6 and TNF-α levels were also upregulated (*P* < 0.001). The C-reactive protein (CRP), rheumatoid factor (RF), erythrocyte sedimentation rate (ESR) and anti-cyclic citrullinated peptide antibody (Anti-CCP) levels in RA patients were generally elevated (*P* < 0.001). NORAD was positively correlated with the levels of clinical indicators and inflammatory factors (*P* < 0.0001). Mechanistically, NORAD may affect the progression of RA by targeting and negatively regulating miR-204-5p.

**Conclusions:**

There is a correlation between NORAD and the processes of RA, and NORAD has the potential to predict and diagnose the occurrence of RA.

## Background

Rheumatoid arthritis (RA) is an autoimmune disease with erosive arthritis as the main symptom. Patients often present with joint stiffness, swelling and pain in the early stage, which may eventually lead to joint deformity and loss of mobility [1, 2]. According to data, RA affects as many as 1.6 billion people worldwide, with an incidence of about 1% [3]. The incidence of RA is 0.32% ~ 0.36% in China, and the proportion of women is much higher than that of men [4]. With the change of lifestyle and the aggravation of aging population, the number of patients with RA has been increasing in recent years. Based on existing treatment methods, the patient’s condition can only be controlled to a certain extent. Therefore, the early implementation of intervention for RA patients is crucial to improve the quality of life.

The application of lncRNAs in diseases has been a research hotspot in recent decades. Although lncRNAs do not have the ability to encode proteins, they may regulate target genes by binding with downstream molecules such as microRNAs (miRNAs), thus participating in the physiological and pathological processes of diseases [5, 6]. LncRNA NORAD (NORAD) considered to be an oncogene in a variety of tumors. For example, NORAD was involved in the treatment of oral squamous cell carcinoma by targeting the miR-577/TPM4 axis [7]. Alternatively, upregulated NORAD was observed to enhance the activity of trophoblast cells in preeclampsia [8]. In the investigation of autoimmune diseases, abnormal NORAD expression was found to be associated with multiple sclerosis patients, which attracted our attention [9]. Therefore, we hypothesized that NORAD may also be a promising factor mediating RA production and progression. Moreover, IL-6 and TNF-α are the key inflammatory markers in the development of RA, which are positively correlated with the severity of the disease [10]. Moreover, Rheumatoid factor (RF) and anti-cyclic citrullinated peptide antibody (Anti-CCP) are also specific factors associated with the diagnosis of RA [11]. Theoretically, all of the above factors can be used for RA identification.

Therefore, this study collected and analyzed the clinical data of RA patients, detected the levels of NORAD and inflammatory factors in the serum of patients, and evaluated the diagnostic value and molecular mechanism of NORAD in RA. This may provide new possibilities for the timely identification and treatment of RA patients.

## Methods

### Enrollment of patients

A total of 77 RA patients admitted to Lishui People’s Hospital from June 2021 to October 2022 and 52 healthy subjects during the same period were enrolled. RA patients were eligible for the following criteria: (1) Patients who had complete clinical data and volunteered to participate in this study under informed conditions. (2) Patients were diagnosed according to the criteria of American College of Rheumatology. (3) Patients had not received anti-rheumatic therapy before the study. (4) Patients with other infectious diseases were excluded. The Ethics Committee of our hospital supervised the whole process of this study (No. 20,201,021), and the clinical indicators of the participants are summarized in Table 1.

**Table 1:** Statistics of clinical indicators of participants

Variables	Participants		*P* value
	Normal (*n* = 52)	RA (*n* = 77)	
Age (years)	46.68 ± 10.39	48.19 ± 9.98	0.396
Gender (Male/Famale)	9/43	12/55	0.797
BMI (kg/m^2^)	24.31 ± 4.57	24.12 ± 3.56	0.792
CRP (mg/L)	3.07 ± 0.39	25.05 ± 7.43	< 0.001
RF (U/mL)	8.28 ± 1.98	111.38 ± 12.37	< 0.001
ESR (mm/h)	8.01 ± 2.97	36.49 ± 8.89	< 0.001
Anti-CCP (U/mL)	2.78 ± 0.86	124.39 ± 11.89	< 0.001

Note: RA, rheumatoid arthritis; BMI, body mass index; CRP, C-reactive protein; RF, rheumatoid factor; ESR, erythrocyte sedimentation rate; Anti-CCP, anti-cyclic citrullinated peptide antibody. (Mean ± standard deviation)

### Collection of serum samples

Venous blood samples (6mL) were collected from RA patients and healthy subjects. After blood centrifugation, the supernatant was extracted, and the resulting serum samples were promptly stored in the refrigerator.

### Selection and culture of cells

Cell Applications (San Diego, USA) supplied us with fibroblast-like synoviocytes (FLSs). DMEM medium (ThermoFisher, USA) was prepared and 10% fetal bovine serum (FBS; Biorad, USA) was added, cells were seeded into the medium and cultured in a 37°C incubator with 5% CO_2_.

### Real-time quantitative PCR assay

Total RNA was extracted from the obtained serum samples by adding TRIzol reagent (Invitrogen, USA), and then reverse-transcribed to cDNA using Primescript Reverse Transcriptase kit (Takara, Japan). The RT-qPCR system was configured with the participation of SYBR Green PCR master mix (ThermoFisher, USA). Three sets of parallel experiments were arranged and the operation was performed in triplicate. GAPDH or U6 was used as the internal reference gene, and date were calculated by the 2^−ΔΔCt^ method for relative expression. The specificity of PCR products was confirmed by the melting curve. The primer sequences used for RT-qPCR were as follows: NORAD forward, 5’-TAGGATACATCTTGGACATGGA-3’ and reverse, 5’-CTAATGAACAAGTCCTGACATA-3’; miR-204-5p forward, 5’-GATCTGGAAGAAGATGGTGGT-3’ and reverse, 5’-GAATTCACAGTTGCCTACAGTAT-3’; GAPDH forward, 5’-GTGCACCTTGGTCCATTTG-3’ and reverse, 5’-GGTGAAGACGCCAGTGGA-3’; U6 forward 5’-TTCGGCAGCACATATACT-3’ and reverse, 5’-GTGCAGGGTCCGAGGTAT-3’.

### Enzyme-linked immunosorbent assay (ELISA)

Serum levels of inflammatory factors (IL-6, TNF-α) were measured in healthy controls and RA patients according to the instructions of the ELISA kit (R&D Systems, USA).

### Luciferase activity reporter assay

Wild-type NORAD (WT) was constructed from the junction sites between NORAD and miR-204-5p, and the mutant-type NORAD (MUT) was obtained through the mutation sites. Control, mimic NC, miR-204-5p mimic and NORAD-WT/MUT were co-transfected into FLSs using lipofectamine 2000 reagent (Invitrogen, USA) and cultured for 48 h. Among them, mimic NC and miR-204-5p mimic were designed and synthesized by RiboBio (Guangzhou, China). Luciferase activity was measured in the Promega system.

### Statistical analysis

Data analysis and mapping were performed using Graphpad Prism 9.0 and SPSS 20.0 software. Measurement data was represented by mean ± standard deviation, and Student’s t-test or one-way ANOVA was used to compare the differences between groups. The diagnostic potential of NORAD in RA was assessed through the ROC curve. Pearson’s correlation analysis was used for the correlation evaluations. *P* < 0. 05 was considered statistically significant.

## Results

### Expression of NORAD in RA serum

**Fig. 1:**
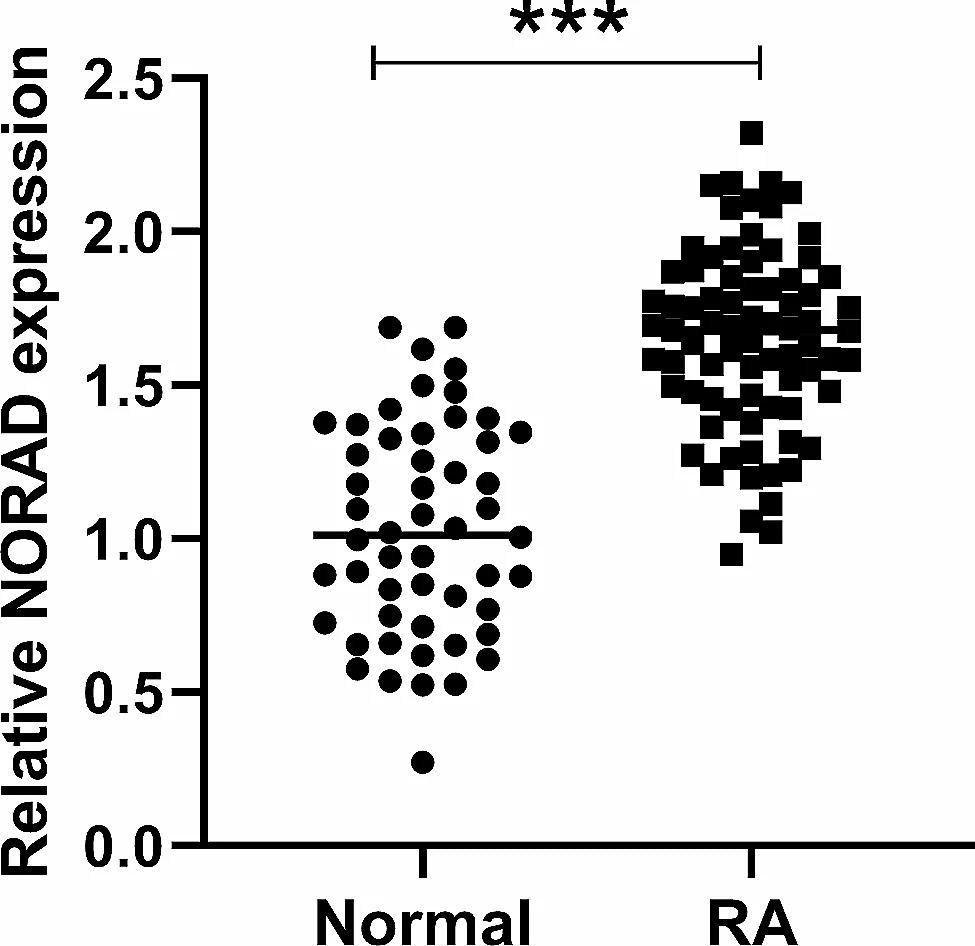
Expression of NORAD in RA serum. Overexpression of NORAD in RA serum was detected by RT-qPCR. ***P < 0.001

Serum NORAD levels were notably higher in RA than in normal samples, as detected by RT-qPCR in Fig. 1 (*P* < 0.001).

### Comparison of clinical features of participants

As stated in Table 1, RA patients differed little from healthy participants in terms of age, gender, and body mass index (BMI), while CRP, RF, ESR and Anti-CCP were obviously elevated in RA patients (*P* < 0.001).

### Correlation between serum NORAD and clinical indicators

**Fig. 2:**
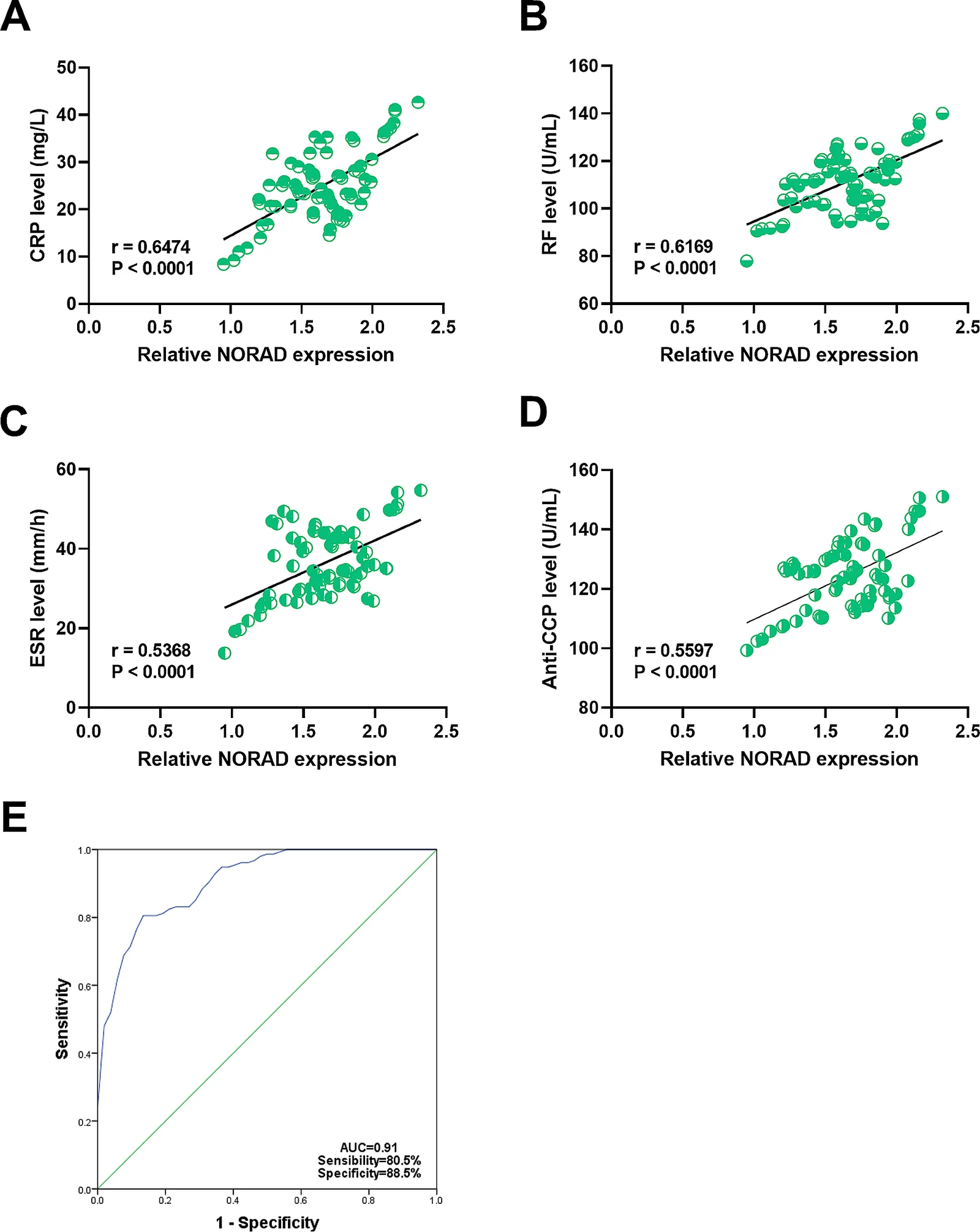
Correlation between Serum NORAD and clinical indicators. (A-D) NORAD expression was positively correlated with CRP (*r* = 0.6474, *P* < 0.0001), RF (*r* = 0.6169, *P* < 0.0001), ESR (*r* = 0.5368, *P* < 0.0001) and Anti-CCP (*r* = 0.5597, *P* < 0.0001). (E) The ROC curve described the potential of NORAD to diagnose RA patients

The relationship between NORAD expression with clinical indicators and inflammatory factors was as follows: NORAD was positively correlated with CRP level (*r* = 0.6474, *P* < 0.0001; Fig. 2A) through Pearson correlation analysis. Meanwhile, the levels of RF (*r* = 0.6169), ESR (*r* = 0.5368) and Anti-CCP (*r* = 0.5597) were also positively correlated with NORAD (*P* < 0.0001; Fig. 2B and D). Based on this, the diagnostic efficacy of NORAD was further evaluated by ROC curve. In Fig. 2E, the AUC of NORAD expression was 0.91, the cut-off value was 1.42 with the high sensitivity (80.5%) and specificity (88.5%), suggesting that NORAD had a good diagnostic value in RA.

### The content of inflammatory factors was upregulated in RA patients

**Fig. 3:**
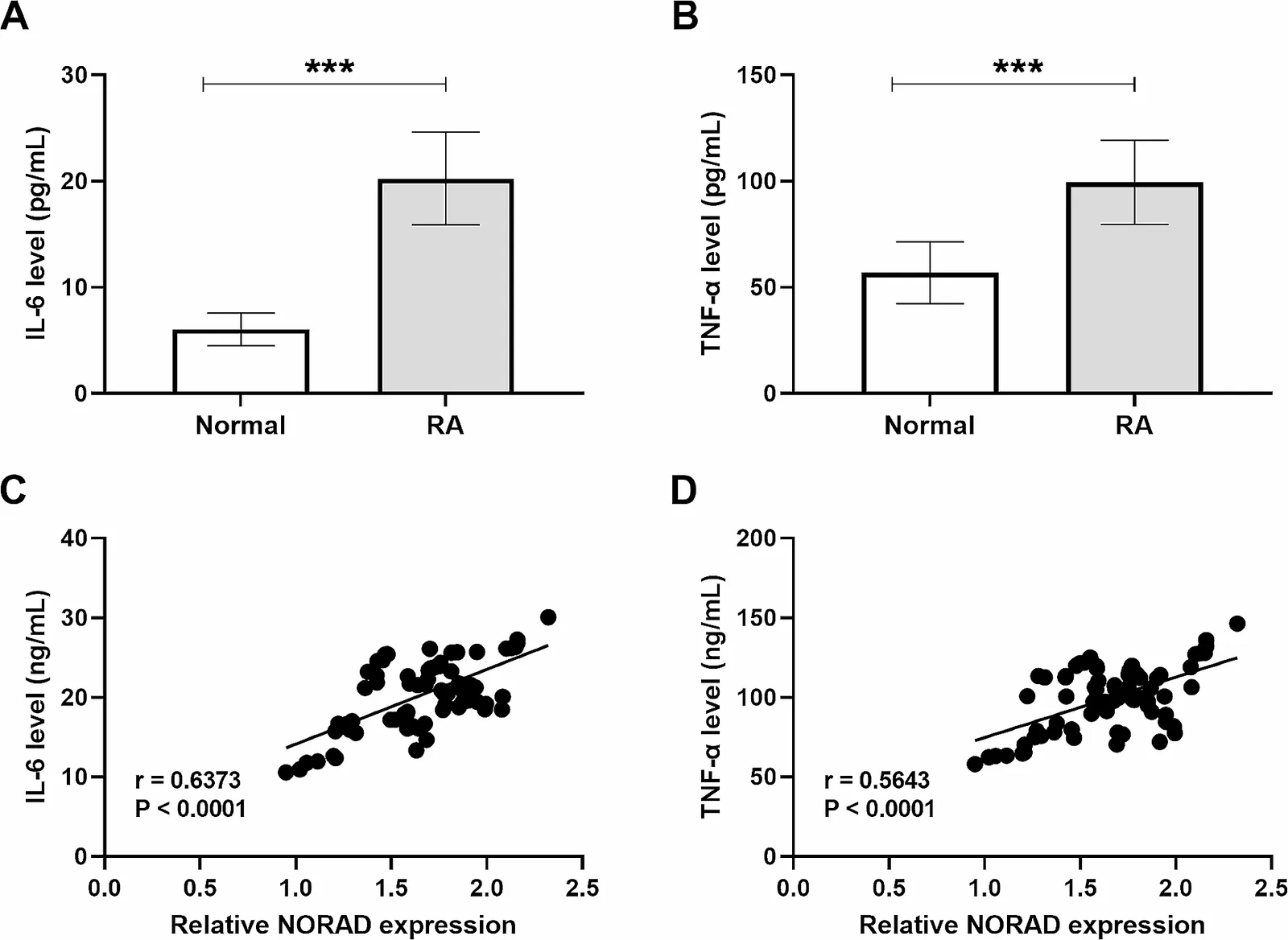
Expression of inflammatory factors and its correlation with serum NORAD. (A-B) ELISA assay revealed increased serum IL-6 and TNF-α levels in RA patients. (C-D) IL-6 (*r* = 0.6373, *P* < 0.0001) and TNF-α (*r* = 0.5643, *P* < 0.0001) levels were directly proportional to NORAD expression. ***P < 0.001

ELISA assay revealed that serum IL-6 (Fig. 3A) and TNF-α (Fig. 3B) levels were upregulated in RA patients compared to normal controls (*P* < 0.001). This implied that a large number of inflammatory factors are produced in patients with RA. Moreover, IL-6 (*r* = 0.6373, *P* < 0.0001) and TNF-α (*r* = 0.5643, *P* < 0.0001) levels were also directly proportional to high NORAD expression in Fig. 3C and D.

### NORAD directly targeted mir-204-5p in RA

**Fig. 4:**
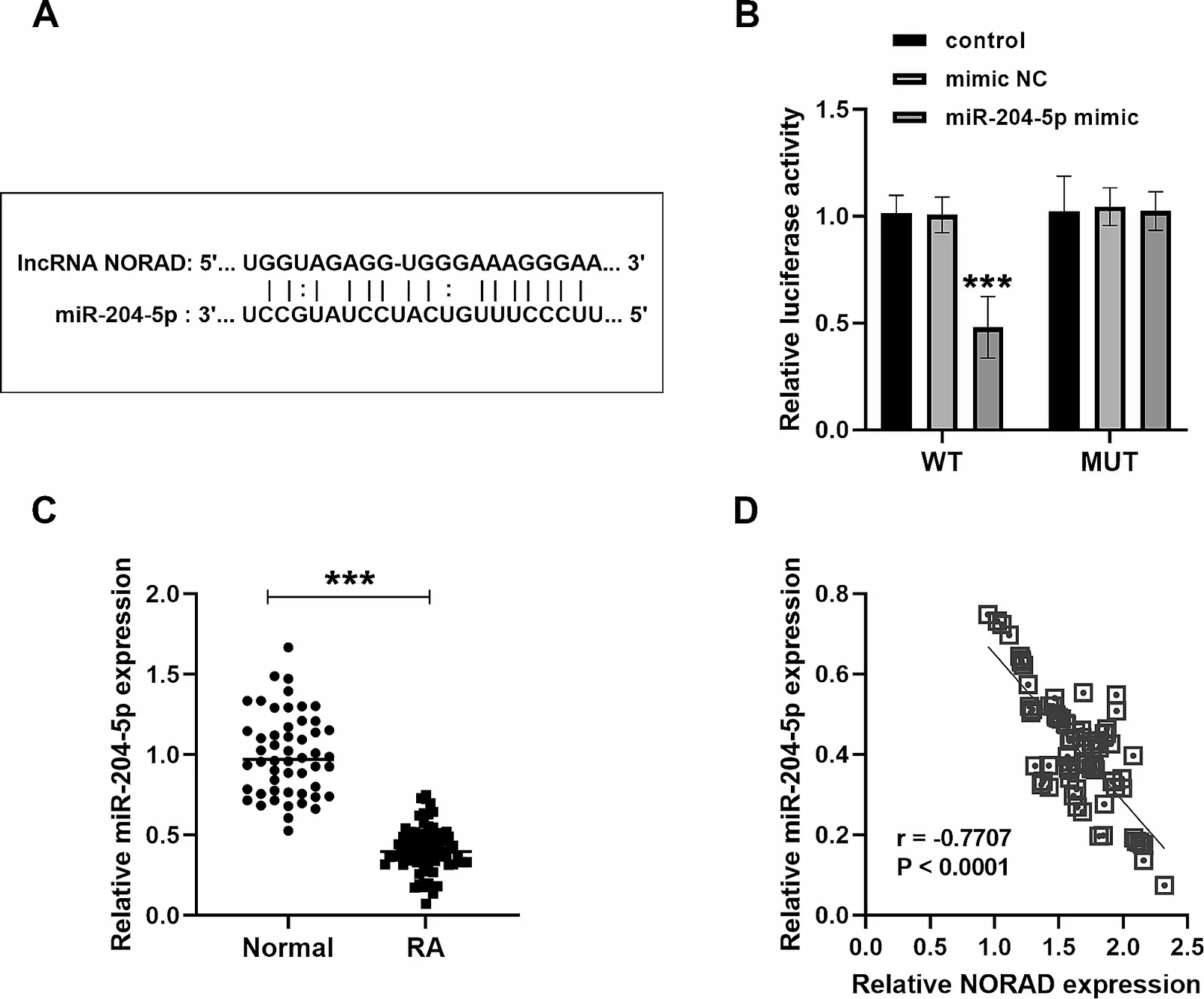
The targeting relationship between NORAD and miR-204-5p. (A) Bioinformatics predicted the existence of complementary bases between NORAD and miR-204-5p. (B) Detection of luciferase activity of NORAD-WT and NORAD-MUT. (C) Serum miR-204-5p was lowly expressed in RA patients. (D) Correlation analysis of serum NORAD and miR-204-5p in RA (*r* = -0.7707, *P* < 0.0001). ***P < 0.001

More deeply, the molecular mechanism of NORAD in RA was investigated. Starbase website (http://www.starbase.sysu.edu.cn) to predict the NORAD and miR-204-5p between complementary bases in Fig. 4A. The targeting relationship between NORAD and miR-204-5p was further verified by luciferase activity assay. According to the results in Fig. 4B, it was observed that the luciferase activity of NORAD-WT decreased after co-transfection with miR-204-5p mimic, while that of NORAD-MUT did not change significantly, indicating that NORAD sponged miR-204-5p. In addition, serum miR-204-5p was down-regulated in RA patients compared with the normal group, as assessed by RT-qPCR (Fig. 4C). Pearson analysis showed that the serum NORAD and miR-204-5p was inversely proportional (*r* = -0.7707, *P* < 0.0001; Fig. 4D), indicating that NORAD negatively regulated the level of miR-204-5p.

## Discussion

RA is a systemic disease whose etiology is still being explored [12]. In clinical practice, RA is often diagnosed by imaging examination and rheumatoid factor detection, but there are still some patients with joint damage at the time of diagnosis, causing irreparable damage [13]. Therefore, the exploitation of sensitive biomarkers is of great significance for the early identification and treatment of RA.

With the in-depth study of lncRNAs in diseases, its relationship with the occurrence and development of RA has been gradually revealed. For example, Bi et al. demonstrated that lncRNA PICSAR was elevated in RA synovial fluid and cells, which affected cell growth and proinflammatory factor production by targeting miR-4701-5p [14]. Zhang et al. proposed that lncRNA GAS5 was upregulated in RA tissues and FLSs, and its specific adsorption of miR-361-5p may play an inhibitory role in the progression of RA [15]. NORAD is located in human chromosome 20q11.23 and includes 5339 nucleotides, which is associated with immune diseases [16, 17]. In our study, NORAD was confirmed to be enhanced in serum of RA and has high diagnostic value, so we believe that NORAD may be a potential biomarker for RA. Furthermore, it was learned that the levels of CRP, RF, ESR and Anti-CCP indicators were obviously increased in RA patients than those in healthy people through the analysis of clinical information from the included samples, which was consistent with previous results [18]. Among them, CRP is an acute phase response protein involved in liver synthesis, and its changes are closely related to the occurrence of RA [19]. RF and Anti-CCP are both autoantibodies, which are auxiliary indicators for clinical diagnosis of RA [20]. ESR is also considered as one of the diagnostic indicators of RA, and its level reflects the severity of the disease [21]. Therefore, this study identified the relationship between the above indicators and NORAD expression, and correlation analysis confirmed that NORAD was positively correlated with the levels of clinical indicators CRP, RF, ESR and Anti-CCP. Meanwhile, IL-6 and TNF-α are common inflammatory cytokines, and their levels are directly proportional to the degree of RA lesions [22, 23]. In the existing evidence, inflammatory factors represented by IL-6 and TNF-α were up-regulated in RA serum [24, 25], which was also confirmed in our study. The inflammatory factors IL-6 and TNF-α was also positively proportional to NORAD expression. It can be concluded that NORAD has diagnostic efficacy for early recognition of RA, and may mediate the progression of RA by regulating the levels of clinical indicators and inflammatory factors. In the exploration of the pathological mechanism of RA, miR-204-5p was confirmed to be the direct target of NORAD. MiR-204-5p was described to aggravate the progression of RA. For example, Wu et al. pointed out that the miR-204-5p level was reduced in plasma exosomes of RA patients [26]. Xiao and colleagues also demonstrated that lncRNA NEAT1 sponge miR-204-5p accelerated the apoptosis of FLSs and controlled the level of inflammatory factors [27]. Similar to the results mentioned, downregulation of miR-204-5p in the serum of RA patients was detected via RT-qPCR method in the present study. Besides, the expression of NORAD was negatively correlated with miR-204-5p in RA. These results indicated that NORAD may interfere with the pathological process of RA by negatively regulating the miR-204-5p level.

## Conclusions

In general, the present study confirmed that serum NORAD and clinical inflammatory factors levels were higher in RA patients than in healthy people, and the prominent expression of NORAD was significantly correlated with clinical indicators and inflammatory factors by assessing the expression of NORAD in RA. NORAD may mediate the progression of RA by regulating the expression of relevant clinical indicators through sponge miR-204-5p. In addition, NORAD has a high diagnostic ability and is expected to become a diagnostic and therapeutic marker for RA. We will further confirm the above conclusions through more complete studies, including the inclusion of more samples, and the addition of cell and animal experiments.

## Data Availability

All data generated or analyzed during this study are included in this article. Further enquiries can be directed to the corresponding author.

## Abbreviations

RA: Rheumatoid arthritis
NORAD: Long non-coding RNA NORAD
RT-qPCR: Real-time fluorescence quantitative polymerase chain reaction
ELISA: Enzyme-linked immunosorbent assay
ROC: Receiver operating characteristic
CRP: C-reactive protein
RF: Rheumatoid factor
ESR: Erythrocyte sedimentation rate
Anti-CCP: Anti-cyclic citrullinated peptide antibody
